# Preparation of Porous Chitosan-Siloxane Hybrids Coated with Hydroxyapatite Particles

**DOI:** 10.1155/2015/392940

**Published:** 2015-05-20

**Authors:** Yuki Shirosaki, Kohei Okamoto, Satoshi Hayakawa, Akiyoshi Osaka, Takuji Asano

**Affiliations:** ^1^Frontier Research Academy for Young Researchers, Kyushu Institute of Technology, 2-4 Hibikino, Wakamatsu-ku, Kitakyushu 808-0196, Japan; ^2^Graduate School of Natural Science and Technology, Okayama University, 3-1-1 Tsushima-naka, Kita-ku, Okayama 700-8530, Japan; ^3^Nikkiso Co., Ltd., Ebisu, Shibuya-ku, Tokyo 150-6022, Japan

## Abstract

This paper describes the apatite deposition of chitosan-silicate porous hybrids derived from chitosan and *γ*-glycidoxypropyltrimethoxysilane (GPTMS) in alkaline phosphate solution. The preparation of porous hybrids with needle-like apatite on their surfaces is described. Following apatite deposition the porous hybrids maintained high porosity. The enzymatic degradation rate was low even after 6 months and the porous hybrids were very flexible and cut easily using surgical scissors.

## 1. Introduction

**Figure 1:**
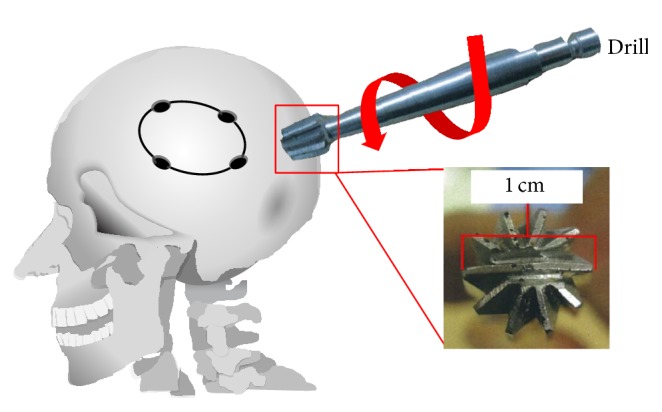
Illustration of craniofacial surgery and use of a drill for the initial hole.

A craniotomy is used to access the brain and can be performed on patients suffering from neurological diseases, traumatic brain injury, brain tumors, hematomas, aneurysms, or a fractured skull. A “burr holes” or “keyhole” is made to allow insertion of a surgical tool, such as a shunt, a drain, an intracranial pressure monitor, or an endoscope or can be used to repair a skull fracture (Figure 1) [1–3].

Following surgery, a “burr hole” must be closed; however current methods, such as hydroxyapatite button [4, 5], titanium plates [6, 7], or bone substitutes, such as calcium phosphate cements (CPCs), have associated risks [8–12]. CPCs may leak into the brain and make contact with cerebrospinal fluid or blood. Many of them also degrade slowly in the body and may cause delayed inflammation [13]. Hydroxyapatite buttons have similar risks and are not flexible so do not complement and therefore “fit” defects on the skull. Furthermore, titanium plates cause thinning of the surrounding soft tissue and extrusion [14, 15] and block observations with MRI. Therefore, new bone substitute materials are required for “burr holes.”

Chitosan-*γ*-glycidoxypropyltrimethoxysilane (GPTMS) hybrid membranes are cytocompatible with the human osteosarcoma cell, MG63, and human osteoblast (HOB) bone marrow cells [16–19]. In the latter case, a fibrillar extracellular matrix with numerous calcium phosphate globules forms [17]. Apatite deposition on the membranes can be improved by releasing calcium ions from the hybrid matrix. The porous scaffold derived from these hybrid membranes is also cytocompatible and apatite can be deposited in the pores [19]. Our group has shown that the rate of biodegradation is low, even in enzymatic solution [20] and such hybrids would remain until the skull has regenerated.

Chitosan-siloxane hybrids must be washed with NaOH solution to neutralize residual acetic acid present in the matrix and it is difficult to control the amount of calcium ions present. Huang et al. [21] soaked calcium-containing borate glass spheres in alkaline phosphate solutions to form the apatite. Hayakawa et al. [22] also used alkaline phosphate solution to fabricate hydroxyapatite nanorods on the silicate glass surface. The chitosan-silicate hybrids also contained calcium ions and silicate networks, and apatite formation would be expected in alkaline phosphate solution following neutralizing.

In this study, the chitosan-silicate porous hybrids were soaked in Na_2_HPO_4_ solution and apatite formation was analyzed. The apatite formation was confirmed and the hybrids were characterized with or without surface apatite deposits.

## 2. Materials and Methods

### 2.1. Preparation of the Porous Hybrids

Chitosan (0.5 g, high molecular weight, deacetylation: 79.0%, Aldrich, USA) was dissolved in aqueous acetic acid (0.25 M, 25 mL). GPTMS (Lancaster, Lancashire, UK) and calcium chloride (Nacalai Tesque, Kyoto, Japan) were added to the chitosan solution to give a molar ratio of chitosan : GPTMS (ChG) (1 : 0.5) or chitosan : GPTMS : CaCl_2_ (ChGCa) (1.0 : 0.5 : 1.0) as shown in Table 1. One mole of chitosan equates one mole of deacetylated amino groups. The mixtures were stirred for 1 h at room temperature and a fraction of each resultant sol was poured into a polystyrene container and frozen at −20°C for 24 h. The frozen hybrids were then transferred to a freeze-dryer (FDU-506, EYELA, Tokyo, Japan) for 12 h until dry. The resultant porous ChG and ChGCa hybrids were then washed with NaOH (0.25 M) and distilled water to neutralize remaining acetic acid and were again lyophilized. Some ChGCa hybrids were not washed with NaOH and they were soaked in aqueous Na_2_HPO_4_ (0.01 M, pH8.8) at 80°C for various fixed periods (ChGCa_*x*d [*x* = soaking time]). The hybrids were then washed with distilled water and again lyophilized. Each sample has 2 mm thickness and 18 mm in diameter.

### 2.2. Structural Characterization and* In Vitro* Biodegradability of the Porous Hybrids

The crystal deposits on the sample surfaces were identified with an X-ray diffractometer equipped with a thin-film attachment (TF-XRD, X'pert-PROMPD, PANalytical, Almelo, Netherlands; CuK*α*, *λ* = 1.5418 Å, 45 kV and 40 mA). The incident beam was fixed at *θ* = 1° and the detector was step-scanned around the 2*θ* axis from 20° to 40° at a rate of 0.02°/step with a count time of 2 s. The morphology or surface microstructure of the porous hybrids was observed using a field emission scanning electron microscope (FE-SEM, S-4800, Hitachi High-Technology, Tokyo, Japan). Outlines of the pore were traced and the maximum diameter was derived using image analysis software (Image J, National Institutes of Health, Bethesda, MD, USA). At least 20 pores were selected from three different areas of each sample. Bulk density was derived from the thickness of the actual and apparent density and the apparent porosity of the hybrids. The porosity was calculated using (1) [23]

(1) Porosity% =Vm−VpVm×100=D×A−mm/rpD×A×100,

where *V*_*m*_ is the total sample volume, *V*_*p*_ is the polymer volume, *r*_*p*_ is the density of chitosan (0.858 g/cm^3^), *A* is the proportion of the sample, *m*_*m*_ is the weight, and *D* is the thickness. The degree of cross-linking was evaluated with a ninhydrin assay and defined as the percentage of free amino groups in the hybrids. Pulverized hybrids were suspended in aqueous acetic acid (0.25 M) for 1 h at room temperature. Ninhydrin solution (ninhydrin reagent L-8500 Set, Wako Chemicals, Osaka, Japan) was added to the suspension and kept at 80°C for 20 min. The optical absorbance of the supernatant was then recorded at 568 nm with an ultraviolet-visible spectrophotometer (UV-2500, Shimadzu Corp., Kyoto, Japan), from which the percentage of free amino groups in the sample was derived [24]. The degree of cross-linking was calculated using

(2) Degree  of  cross-linking =1−A568  of  each  samplesA568  of  chitosan×100.

The porous hybrids were soaked in phosphate-buffered saline solution (PBS, pH 7.4) to determine water uptake, that is, the amount of water adsorbed into the porous hybrids according to (3). *W*_*d*_ and *W*_*w*_ stand for the weights before and after PBS soaking, respectively. Consider the following:

(3) $$\begin{array}{c} Water\;uptake\;\left(\%\right)=\frac{100\;\left(W_{w}-W_{d}\right)}{W_{d}}. \end{array}$$

To examine* in vitro* biodegradability, the porous hybrids were soaked in 1 mg/mL lysozyme solution (PBS, pH 7.4). The lysozyme solution was changed each 3 days. The weight loss of the porous hybrids was calculated according to (4), where *W*_*b*_ and *W*_*a*_ stand for the weights before and after being soaked in lysozyme solution, respectively. Consider the following:

(4) $$\begin{array}{c} Weight\;loss\;\left(\%\right)=\frac{100\left(W_{b}-W_{a}\right)}{W_{b}}.\; \end{array}$$

## 3. Results and Discussion

**Figure 2:**
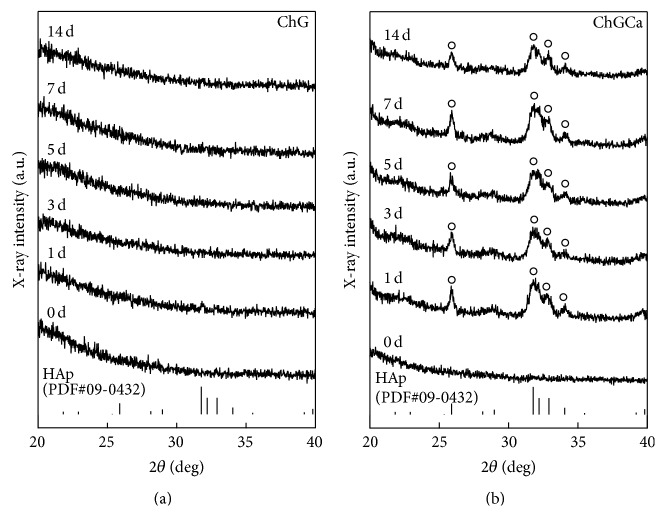
TF-XRD patterns for ChG and ChGCa before and after soaking in 0.01 M Na_2_HPO_4_ solution for various periods. ^○^HAp.

ChG and ChGCa were soaked in 0.01 M Na_2_HPO_4_ for 14 days. And the TF-XRD patterns are shown in Figure 2. Apatite deposits were not detected on ChG but were detected on ChGCa after 1 day. In the case of ChGCa, the pH of the solution decreased from 8.8 to 7.4 because of residual acetic acid used for the preparation of chitosan (sample that was not washed with NaOH solution before it was soaked in aqueous Na_2_HPO_4_). After soaking in Na_2_HPO_4_ solution, the acetic acid and calcium chloride released into the solution and calcium ions reacted with phosphate ions in Na_2_HPO_4_ to form apatite. In this study and in previous work [18, 19], EDX analysis indicated the presence of Si in all the hybrids (ChG, ChGCa, and ChGCa_*x*d). It means that the silanol groups or siloxane networks exist in the hybrids. The presence of silanol groups on the ChGCa was available to the apatite nuclei [19] and a physiological pH provides suitable conditions for apatite formation and growth. After 3 days, the apatite growth stopped, indicating that release of calcium ions was immediate but momentary.

**Figure 3:**
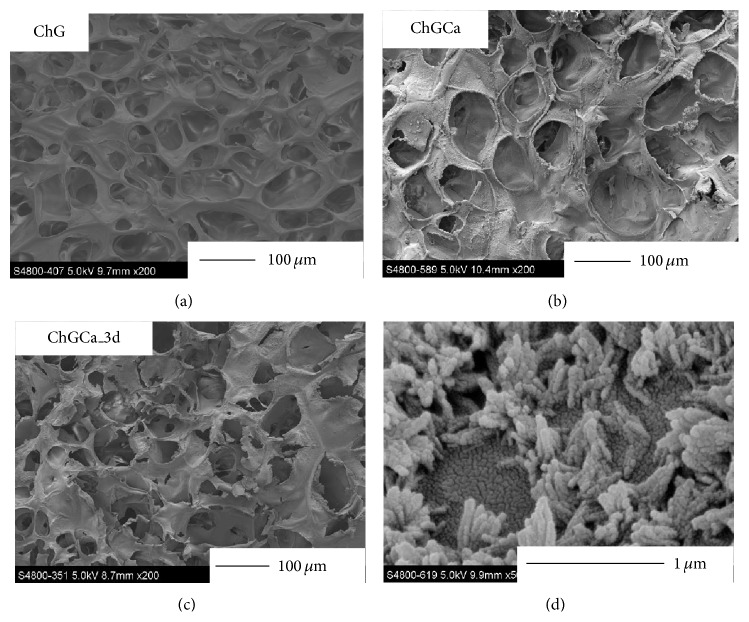
FE-SEM images of cross-section for the ChG, ChGCa, and ChGCa_3 d. The image at lower right is magnification of ChGCa_3 d.

Figure 3 shows FE-SEM images of cross-section for the hybrids. In the case of SBF (simulated body fluid, Kokubo solution) [19], spherical deposits covered the surface of ChGCa. In this work, the apatite deposits on the ChGCa in Na_2_HPO_4_ solution were needle-like (d). Apatite also deposited inside of the hybrids regardless of the depth. Calcium ions diffused from the hybrid pore surface and phosphate ions can react only near the interface between the pore surface and solution. Apatite can grow following the diffusion of calcium ions because they are not present in solution and the shape became needle-like.

The chitosan-GPTMS hybrids (both ChGCa and ChG) have interconnected pores and this is consistent with precious reports [18, 19]. After soaking in 0.01 M Na_2_HPO_4_, the porous microstructure was maintained.  Table 2 shows the pore size, porosity, and degree of cross-linking. The pore size for ChGCa (69.8 ± 13.4 *μ*m) and ChGCa_3 d (62.1 ± 12.5 *μ*m) was slightly higher than that for ChG (48.8 ± 16.9 *μ*m). This is because washing with aqueous NaOH or soaking in Na_2_HPO_4_ solution causes swelling and an enlargement of the pores. As previously reported [18], ChG and ChGCa have high porosity, with 93.8 ± 0.2% and 92.4 ± 0.4%, respectively. After soaking in Na_2_HPO_4_ solution, this porosity did not change and was 94.8 ± 0.3% for ChGCa_3 d. The degree of cross-linking for the porous hybrids was lower than that for solid membrane even with the same composition [17, 18]. As shown in Table 2, the degree of cross-linking for ChGCa (28.7 ± 4.9%) and ChGCa_3 d (31.3 ± 3.9%) is slightly lower than that for ChG (36.6 ± 4.0%). This is because calcium ions interacted with amino groups in the chitosan matrix [16], so cross-linking is inhibited.

**Figure 4:**
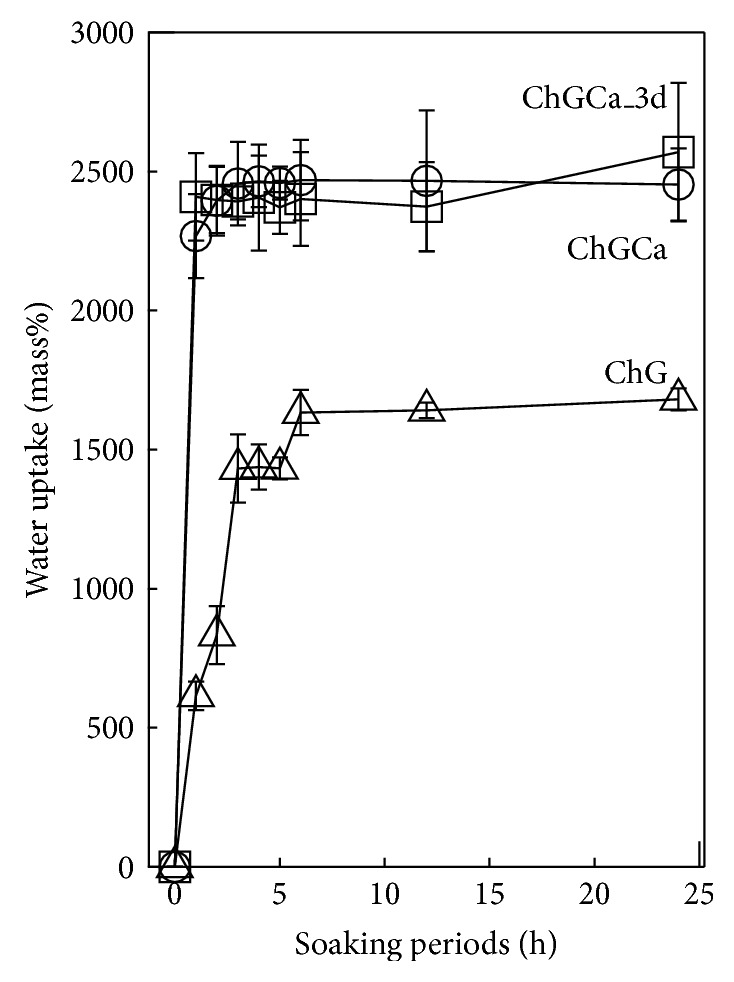
Water uptake of ChG, ChGCa, and ChGCa_3 d after soaking in PBS for 1 day.

Water uptake in PBS is shown in Figure 4. A plateau or equilibrium is observed for ChGCa and ChGCa_3 d after 2 h and after 5 h for ChG. After soaking in PBS, the shape of the porous hybrids did not change. ChGCa and ChGCa_3 d absorbed 1.5 times more PBS than did ChG. ChGCa has more space for water retention inside the hybrids than does ChG even with apatite deposits on the surface (ChGCa_3 d). On the other hand, ChGCa may release calcium ions into PBS and exchange or react with PBS resulting in high water uptake.

To investigate the biodegradability, the porous hybrids were soaked in lysozyme solution.  Table 3 shows the resultant loss in weight. After 1 month, the weight of ChGCa decreased by approximately 50%, while ChG and ChGCa_3 d scarcely changed. This indicates that the release of calcium ions from ChGCa contributes to the weight loss because they do not deposit apatite in lysozyme solution. The ChGCa_3 d scarcely degraded after 1 month and then by approximately 40% within 3 months. Calcium ions interacted with silanol groups derived from GPTMS and the condensation of GPTMS, that is, the formation of a Si-O-Si network, was inhibited by adding calcium ions. The silanol groups in ChGCa were used to deposit the apatite in Na_2_HPO_4_ and the hybrid did not degrade in 1 month in lysozyme solution. However, after a long period of soaking (3–6 months), they started to degrade faster than ChG. ChGCa and ChGCa_3 d absorb more PBS than does ChG and the enzyme can work in the pores and the matrix. Moreover, ChGCa released calcium ions into lysozyme solution and reduced their weight. Anyway, the enzymatic degradation of a chitosan-silicate hybrid matrix is slow; therefore, this material is suitable for the regeneration of skull.

## 4. Conclusion

The chitosan-GPTMS porous hybrids deposited apatite in alkaline phosphate solution. This means that the calcium ions in the hybrids can be used to form the apatite without loss during washing. The hybrids had high porosity and water uptake property was maintained after apatite deposition. It is expected that the chitosan-GPTMS porous hybrids coated hydroxyapatite can be compatible to the bone defect.

## Figures and Tables

**Table 1:** Starting composition of the hybrids (molar ratio).

Sample	Chitosan	GPTMS	CaCl_2_
ChG	1.0	0.5	0
ChGCa	1.0	0.5	1.0

**Table 2:** Pore size, porosity, and degree of cross-linking for ChG, ChGCa, and ChGCa_3 d.

Sample	Pore size (*μ*m)	Porosity (%)	Degree of cross-linking (%)
ChG	48.8 ± 16.9	93.8 ± 0.2	36.6 ± 4.0
ChGCa	69.8 ± 13.4	92.4 ± 0.4	28.7 ± 4.9
ChGCa_3 d	62.1 ± 12.5	94.8 ± 0.3	31.3 ± 3.9

**Table 3:** Weight loss (%) of ChG, ChGCa, and ChGCa_3 d after being soaked in 1 mg/mL lysozyme/PBS for various periods at 36.5°C.

Sample	Soaking period (month)		
	1	3	6
ChG	1.01 ± 1.43	14.87 ± 5.46	28.11 ± 2.99
ChGCa	49.78 ± 9.59	56.56 ± 1.02	65.27 ± 1.69
ChGCa_3 d	2.16 ± 0.75	40.97 ± 5.92	46.23 ± 3.92
